# Are floating algal mats a refuge from hypoxia for estuarine invertebrates?

**DOI:** 10.7717/peerj.3080

**Published:** 2017-03-23

**Authors:** Michael R.S. Coffin, Kyle M. Knysh, Emma F. Theriault, Christina C. Pater, Simon C. Courtenay, Michael R. van den Heuvel

**Affiliations:** 1 Canadian Rivers Institute at the Department of Biology, University of Prince Edward Island, Charlottetown, Prince Edward Island, Canada; 2 Canadian Rivers Institute at the School of Environment, Resources and Sustainability, University of Waterloo, Waterloo, Ontario, Canada

**Keywords:** Eutrophication, Hypoxia, Community ecology, Anoxia, Ulva, Ephemeral algae, Amphipod, Estuary, Invertebrate

## Abstract

Eutrophic aquatic habitats are characterized by the proliferation of vegetation leading to a large standing biomass that upon decomposition may create hypoxic (low-oxygen) conditions. This is indeed the case in nutrient impacted estuaries of Prince Edward Island, Canada, where macroalgae, from the genus *Ulva,* form submerged ephemeral mats. Hydrological forces and gases released from photosynthesis and decomposition lead to these mats occasionally floating to the water’s surface, henceforth termed floating mats. Here, we explore the hypothesis that floating mats are refugia during periods of sustained hypoxia/anoxia and examine how the invertebrate community responds to it. Floating mats were not always present, so in the first year (2013) sampling was attempted monthly and limited to when both floating and submerged mats occurred. In the subsequent year sampling was weekly, but at only one estuary due to logistical constraints from increased sampling frequency, and was not limited to when both mat types occurred. Water temperature, salinity, and pH were monitored bi-weekly with dissolved oxygen concentration measured hourly. The floating and submerged assemblages shared many of the same taxa but were statistically distinct communities; submerged mats tended to have a greater proportion of benthic animals and floating mats had more mobile invertebrates and insects. In 2014, sampling happened to occur in the weeks before the onset of anoxia, during 113 consecutive hours of sustained anoxia, and for four weeks after normoxic conditions returned. The invertebrate community on floating mats appeared to be unaffected by anoxia, indicating that these mats may be refugia during times of oxygen stress. Conversely, there was a dramatic decrease in animal abundances that remained depressed on submerged mats for two weeks. Cluster analysis revealed that the submerged mat communities from before the onset of anoxia and four weeks after anoxia were highly similar to each other, indicating recovery. When mobile animals were considered alone, there was an exponential relationship between the percentage of animals on floating mats, relative to the total number on both mat types, and hypoxia. The occupation of floating mats by invertebrates at all times, and their dominance there during hypoxia/anoxia, provides support for the hypothesis that floating mats are refugia.

## Introduction

Coastal watersheds and estuaries are vulnerable to impact from anthropogenic activity as human population density tends to be higher near the coast (Larkum, Orth & Duarte, 2006; Lotze et al., 2006; Short et al., 2006). Historically, many temperate estuaries were oligotrophic and dominated by seagrasses (Cooper & Brush, 1993; Larkum, Orth & Duarte, 2006; Short et al., 2006; Burkholder, Tomasko & Touchette, 2007). Nutrient pollution from human sources has led to dramatic changes to estuarine and coastal habitats including displacement of seagrass by macro- and microalgae (Burkholder, Tomasko & Touchette, 2007). Seagrass-dominated systems typically have higher diversity of fish and invertebrates than macroalgal habitat, (Baird et al., 2004; Cardoso et al., 2004a; Cardoso et al., 2004b), partially due to greater spatial complexity, yet many of the species common to seagrass persist in macroalgae (Olsen et al., 2013). Given that many impacted systems are unlikely to be restored in the short or medium term, establishing what, if any (Antón et al., 2011), ecosystem services are provided by eutrophic habitats has been a focus of recent research (Duarte, 2000; Lyons et al., 2014). Although eutrophication has been well-studied, current understanding of community responses to hypoxia, here defined as <2 mg/L, has been limited by: the ability to predict and document hypoxic events (Middelburg & Levin, 2009); changes to faunal behaviours when exposed to hypoxia (Riedel et al., 2014); and, the subsequent interactions of fauna.

One of the most common characteristics of eutrophication in estuaries is low dissolved oxygen concentration (Howarth et al., 2011). Many algal species can uptake nitrogen more efficiently than seagrasses (Valiela et al., 1997; Hemminga & Duarte, 2000) and in high nutrient environments they can proliferate rapidly, eventually smothering seagrass (Valiela et al., 1997a; Burkholder, Tomasko & Touchette, 2007). This change in primary production results in fluctuating dissolved oxygen as photosynthesis raises dissolved oxygen during the day and cellular respiration in the absence of photosynthesis lowers it at night (Hemminga & Duarte, 2000; Cardoso et al., 2004a). In situations of nutrient excess, a cycle develops whereby algae grow until they shade themselves, die and decompose (Valiela et al., 1997; Burkholder, Tomasko & Touchette, 2007). Decomposition of algae further depresses dissolved oxygen, eventually resulting in hypoxia/anoxia and the development of toxic hydrogen sulphide and other stressors, e.g., ammonia (Valiela et al., 1997; Berezina, 2008). Sustained hypoxia or anoxia can have a catastrophic effect on the local faunal community with animals that are incapable of emigrating being at greatest risk (Ekau et al., 2010; Lyons et al., 2014; Tweedley et al., 2016).

Estuaries in the Southern Gulf of St. Lawrence, Canada, are relatively small and shallow with a mix of semi-diurnal and diurnal tides (Pingree & Griffithis, 1980) and are typically ice-covered from January to mid-April (Manson, Davidson-Arnott & Ollerhead, 2016). Throughout the year, much of the estuary is used for commercial purposes including mussel and oyster suspension- and bottom-aquaculture (Dumbauld, Ruesink & Rumrill, 2009; Skinner, Courtenay & McKindsey, 2013) as well as fishing for American eel (*Anguilla rostrata* Lesueur, 1821), herring (*Alosa* spp.) and Atlantic silverside (*Menidia menidia* (Linnaeus, 1766)), among others. Although nitrogen inputs from human waste and industry are low, nutrient inputs from agriculture can be extremely high, particularly in Prince Edward Island (PEI) (Danielescu & MacQuarrie, 2011; Schein et al., 2012; Bugden et al., 2014). Here, eutrophication manifests as the familiar gradient from inner algae- to outer seagrass-dominated habitat (Valiela et al., 1997; Deegan, 2002; Larkum, Orth & Duarte, 2006; Burkholder, Tomasko & Touchette, 2007), with many sites experiencing intermittent and sustained seasonal hypoxia/anoxia (concentrations of dissolved oxygen <2 mg/L and 0 mg/L, respectively (Bugden et al., 2014)) often lasting days. While a variety of factors and mechanisms contributing to sustained hypoxia have been well-discussed previously (D’Avanzo & Kremer, 1994; Valiela et al., 1997; Touchette & Burkholder, 2000; Burkholder, Tomasko & Touchette, 2007; Heck & Valentine, 2007; Castro & Freitas, 2011; Cebrian, Corcoran & Lartigue, 2013), poor flushing (i.e., high water residence time) is a key factor leading to the development and persistence of hypoxia (Valiela, 1995; Valiela et al., 1997; Hemminga & Duarte, 2000; Burkholder, Tomasko & Touchette, 2007). Estuaries on the north and west coasts of PEI are microtidal and thus more susceptible to sustained hypoxia than estuaries on the south and east coasts that have greater tidal amplitude, although hypoxia can also occur there (Pingree & Griffithis, 1980; Bugden et al., 2014; Tweedley et al., 2016).

The dominant macroalgae (primarily *Ulva lactuca* L. but also *U. intestinalis* L. and *U. linza* L., henceforth referred to as *Ulva* spp.) occur year round, typicallly reaching its maximum density in June–July (Schein et al., 2012). Although *Ulva* spp. have holdfasts, here they typically grow unattached in large sheets (up to ∼0.5 m^2^) and form ephemeral submerged mats. Consequently, hydrodynamic forces dictate mat distribution within an estuary. *Ulva* spp. tend to accumulate in deeper water, where tidal fluctuation has less impact, but still reach high abundances in the relatively placid shallows of the upper estuary that are more uniform in depth. At times, submerged *Ulva* spp. mats rise to the water’s surface, presumably due to tidal forces and/or gases released from sediment and decomposing algae, and become floating mats. There is very little rocky substrate in most PEI upper estuaries, and therefore, in nutrient impacted estuaries, *Ulva* spp. constitute the primary structural habitat. As a result, the majority of fauna live in the interstitial areas among algae fronds and not the loose and often anoxic sediments (Nestlerode & Diaz, 1998; Riedel et al., 2014, M Coffin, pers. obs., 2013).

To our knowledge most studies that examine ephemeral mats typically focus on submerged mats and only rarely on their vertical distribution within the water column (Highsmith, 1985; Salovius, Nyqvist & Bonsdorff, 2005; Kraufvelin et al., 2006). While rafting is a common phenomenon (Thiel & Gutow, 2005) it is unclear if floating mats in estuaries are occupied by the same taxa that occupy submerged mats, by neustonic species, or if they are occupied at all. Furthermore, given that hypoxia/anoxia is typically most severe closer to the substrate and dampened by air-water exchange near the water’s surface it stands to reason that floating mats may be refugia under certain conditions. Here, we examine these questions through field surveys at seven PEI estuaries for fauna on floating and submerged mats in conjunction with hourly monitoring of dissolved oxygen in 2013. Using the same methodology in the subsequent year, but with increased sampling intensity and at a single site, a natural experiment occurred in which sampling was conducted before, during and after a hypoxic/anoxic event.

## Materials and Methods

### Site description and general study design

**Figure 1:**
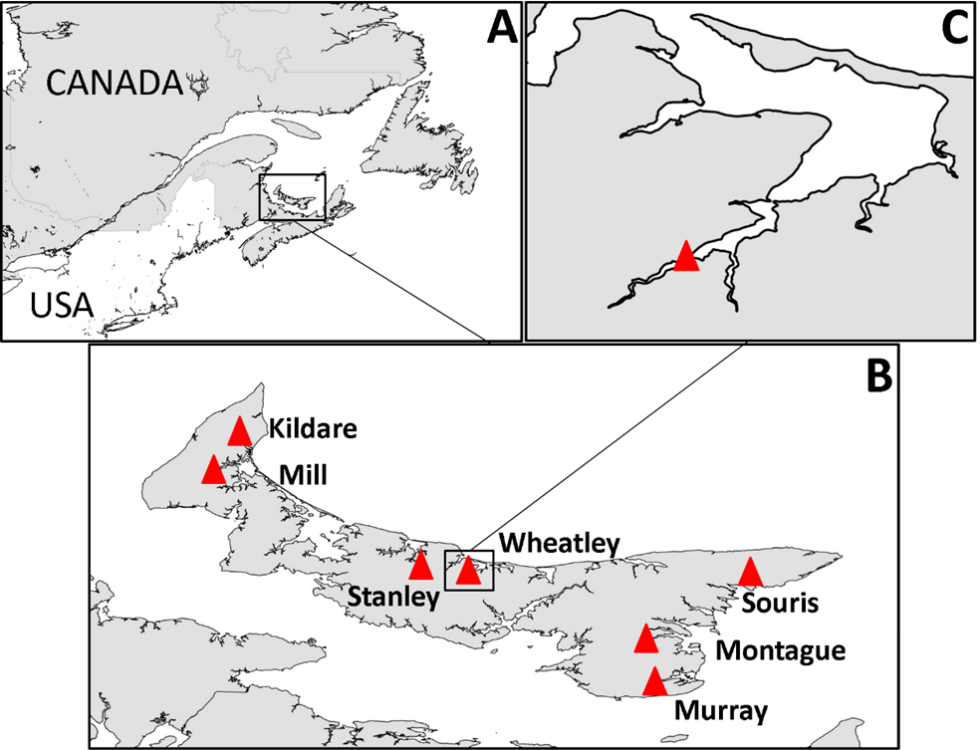
Map of study area. (A) Prince Edward Island in context of eastern North America, (B) PEI estuaries that were sampled indicated by a red triangle and labeled, (C) Logger and sampling location within Wheatley River estuary, the site of the natural experiment.

PEI has short rivers, the baseflow of which is almost entirely from groundwater (Jiang & Somers, 2009; Jiang, Zebarth & Love, 2011). According to the definition used in the Coastal and Marine Ecological Classification Standard (Glibert et al., 2010), estuaries on the north coast of PEI are typically lagoon-type, with barrier islands, and those on the south coast (Northumberland Strait) are coastal embayments and have greater tidal exchange. As there is relatively low freshwater input into PEI estuaries, water is well mixed throughout the water column (Bugden et al., 2014) and saline up most of the estuary to the point where it rapidly becomes fresh; the transition zone from 0–15 practical salinity units (PSU) is a small area. Consequently, there is a stark shift from fresh water to estuarine habitat, and it is this area, at the head of the estuary, that tends to be dominated by *Ulva* spp. at nutrient-impacted sites. In total seven nutrient-impacted estuaries, were selected for study in 2013 with one of those, Wheatley River, studied exclusively in 2014 (Fig. 1).

Onset Hobo^®^ Dissolved Oxygen loggers that recorded dissolved oxygen (mg/L) and water temperature (°C) hourly were deployed within the uppermost areas of estuaries where *Ulva* spp. densities are high and average salinity ranged from 15–20 PSU (Fig. 1C and Table 1 for summary of physicochemical variables). Loggers were deployed 0.5 m from the substrate and therefore not enveloped with algae (in approximately 1–1.5 m of water at mean low tide). Loggers were downloaded bi-weekly and water temperature, salinity, and pH were recorded using a YSI V2 6600 multi-parameter sonde (Yellow Springs, OH, USA) at the same depth and location as the dissolved oxygen logger and also at 0.5 m below the water’s surface. Water chemistry variables were averaged over the course of sampling (*n* = 6; see Table 1). Logger data from the 48 h period prior to invertebrate sampling were used for analyses herein, this threshold was chosen as it is long enough to impact survival and behaviours (Miller, Poucher & Coiro, 2002; Landman, van den Heuvel & Ling, 2005; Vaquer-Sunyer & Duarte, 2008).

**Table 1:** Site characteristics and water chemistry information. Site characteristics and average water chemistry measurements for each site over the course of sampling. Water chemistry values (salinity, pH and dissolved oxygen (mg/L)) were averaged over the entire sampling season (*n* = 6) from 0.5 m above the substrate, i.e., where the logger was located, and additionally 0.5 m below the water’s surface for dissolved oxygen only. Watershed area and N-loading are courtesy of the Prince Edward Island provincial government and based on 2010 land-use layers. Pressure loggers were deployed in summer 2015 at the same locations as the dissolved oxygen loggers. Variability is presented as ± 1 S.E. for water chemistry values.

Site	Sampling times	Watershed area (km^2^)	N-Loading (kg/ha/yr)	Mean tidal amplitude (m)	Salinity	pH	Average DO mg/L (bottom/top)
Kildare	2	5.54	21.12	0.33	21.7 ± 0.5	7.54 ± 0.04	6.5 ± 0.9 / 9.5 ± 1.6
Mill	1	11.65	16.15	0.47	23.1 ± 0.5	7.52 ± 0.05	5.9 ± 1.0 / 8.3 ± 2.3
Montague	1	19.65	10.67	0.82	23.2 ± 0.9	7.49 ± 0.05	5.5 ± 0.4 / 5.9 ± 0.7
Murray	2	7.11	5.37	0.83	24.0 ± 1.0	7.58 ± 0.05	5.9 ± 0.8 / 9.0 ± 1.2
Souris	2	4.85	10.82	0.68	24.6 ± 0.9	7.57 ± 0.08	8.0 ± 0.8 / 9.2 ± 1.9
Stanley	2	8.64	10.65	0.39	24.2 ± 0.6	7.58 ± 0.04	6.0 ± 0.7 / 7.2 ± 1.7
Wheatley	3	6.14	12.64	0.44	24.4 ± 0.4	7.64 ± 0.04	7.2 ± 0.4 / 8.6 ± 1.3
Wheatley (2014)	6	6.14	12.64	0.44	24.0 ± 0.3	7.91 ± 0.03	8.4 ± 0.6 / 8.6 ± 0.6

Invertebrate sampling in 2013 was attempted monthly (June–August) at all sites but was only accomplished if surface floating mats were present at the time of sampling (Fig. 1 and Table 1). On each sampling occasion five replicate samples were taken for submerged mats and three replicate samples for floating mats. To better account for temporal changes in community response to hypoxia, the experimental design was altered in the following year. All sampling in 2014 occurred at Wheatley River with five replicate samples taken each week (June 23–July 28) for submerged mats and five replicate samples for floating mats if they occurred (four of the six sampling times). Animals were sampled under scientific license granted by the Department of Fisheries and Oceans, Canada (SG-RHG-13-006; SG-RHG-14-006).

### Invertebrate sampling

Locations of 1–2 m depth selected randomly within a 200 m radius of the dissolved oxygen logger (Fig. 1C) were sampled from a boat between slack and high tide (0.33–0.83 m). Preliminary sampling revealed that water-column hypoxia occurred at spatial scales that exceeded our sampling region and was not restricted to macroalgae patches (MRS Coffin, 2013, unpublished data). Submerged mats were sampled randomly by traveling in an arbitrary direction and attempting collection after mooring. For floating mat collection, the first mat encountered on a random transect was sampled, with the exception of very small floating mats or cases in which the mat was immediately adjacent to a previously sampled floating mat. Floating mats were not always present, but when they occurred they were abundant and distributed evenly. Since floating mats were quite mobile, no attempt was made to collect submerged mats from the same location as floating mats. Both mat types were collected using two bow-head garden rakes (∼40.6 cm width with 2.5 cm between tines). The sampler would place the rakes, one in each hand and shoulder width apart (a sampling area of approximately 0.25 m^2^), just above the substrate for submerged mats and just below the vegetation for floating mats, and bring them together underwater. Samples were then brought into the boat and into a bucket of macroinvertebrate-free water. Algal samples for submerged and floating mats were relatively large and had similar dry weights (Dry weights are presented as the overall mean ± 1 standard error. In 2013: 23.9 ± 2.9 g and, 25.7 ± 2.0 g for submerged (*n* = 65) and floating (*n* = 41) mats, respectively; in 2014: 31.2 ± 2.4 g and 26.5 ± 2.8 g for submerged (*n* = 30) and floating (*n* = 20) mats, respectively). Although some invertebrates on the outside of the algal sample may have been lost prior to entry into the boat, the majority of the sample was packed tightly together, preventing animals from escaping. *Ulva* spp. blades were manually separated from invertebrates, placed in a plastic bag and brought to the laboratory where they were frozen (−20 °C) for storage. Algal health was not assessed directly, but only algae that appeared living was retained. The invertebrates, water, and sediment slurry was sieved in the field using 500 µm mesh and stored in a snap-seal plastic container in 95% ethanol until processing.

All samples were further processed in the laboratory using a dissecting microscope (40X magnification). Vegetation was thawed and then dried at 60 °C for 48 h and weighed. Samples were standardized by the dry mass (DM) of *Ulva* spp. and therefore invertebrates are presented as individuals per g DM (henceforth, individuals/g) of *Ulva* spp. Macroinvertebrates were identified to a practical level for comparison using the dissecting microscope and appropriate taxonomic guides (Triplehorn & Johnson, 2005; Bousfield, 1973; Appy, Linkletter & Dadswell, 1980; Pollock, 1998; Merritt, Cummins & Berg, 2008; Thorp & Covich, 2010). Taxa were generally identified to family, with characteristic lower classification such as genera and taxon noted (see Table 2).

**Table 2:** Colour coded list of species found in each habitat type and study site. List of all taxa found at every estuary studied. Animals found in Wheatley were combined for both field seasons. Mat type is defined by colored cells where blue cells containing the letter ‘S’ represent taxa found on submerged mats only, yellow cells containing the letter ‘F’ represent taxa found only on floating mats, and green cells containing the letter ‘B’ represent taxa found in both habitats. Mobile taxa are indicated with an asterisk (*).

Species	Kildare	Mill	Stanley	Wheatley	Souris	Montague	Murray
**Mollusca-Gastropoda**							
Nassariidae: *Nassarius obsoletus* (Say, 1822)	F	S	B	B		B	B
Hyrdobiidae	B	B	B	B	B	B	B
Cerithiidae	S		S	B			
Pyramidellidae	S			B			
Cylichnidae: *Acteocina* sp.	S			F			S
Columbellidae: *Astyris lunata* (Say, 1826)		S		B			
Littorinidae: *Littorina* spp.	F			B		B	B
Marginellidae				S			
**Mollusca-Bivalvia**							
Myidae: *Mya arenaria* (Linnaeus, 1758)	B	B	S	S	S		F
Veneridae: *Gemma gemma* (Totten, 1834)	S	S		S	F	S	F
Mytilidae	B	B	B	S	S		B
Ostreidae: *Crassostrea virginica* (Gmelin, 1791)		S					
**Annelida-Polychaeta**							
Nereidae	B	B	B	B			S
Capitellidae	B	F	S	B			B
Nephtyidae: *Nephtys* spp.	S	S	S	S			S
Orbiniidae		S	S	S			S
Spionidae	S		S	S			S
Pectiniridae	S	S					
Terebellidae							S
Polynoidae	S	S		S			
Serpulidae: *Spirorbis spirorbis* (Linnaeus, 1758)							F
**Annelida-Clitellata**							
Naididae	F	F		F		S	F
**Arthropoda-Crustacea-Amphipoda**							
* Gammaridae: *Gammarus mucronatus* (Say, 1818)	B	B	B	B	B	B	B
* Gammaridae: *G lawrencianus* (Bousfield, 1956)	B	B	B	B	B	F	B
* Gammaridae: *G oceanicus* (Segerstråle, 1947)	F	S	F	S		B	S
* Ampithoidae					F	F	
* Corophiidae	B	B	B	B	B		B
**Arthropoda-Crustacea-Isopoda**							
* Janiridae: *Jaera* spp.			S		B	B	
**Arthropoda-Crustacea-Decapoda**							
Palaemonidae: *Palaemon* spp.				S			F
Crangonidae: *Crangon septemspinosa* Say, 1818						S	
**Arthropoda-Crustacea-Tanaidacea**							
Leptocheliidae			S				
**Arthropoda-Crustacea-Branchiura**							
Argulidae: *Argulus* sp.	F						
**Arthropoda-Crustacea-Cirripedia**							
*Semibalanus balanoides*	F						
**Arthropoda-Hexapoda-Insecta**							
**Insecta-Diptera**							
* Chironomidae: Orthocladiinae	F	F	B	F		F	
* Chironomidae: Chironominae: Chironomini	B	S		S	F	F	S
Ephydridae	F		F	F	F	F	F
Ceratopogonidae					F		
Tipulidae (*sensu lato*)					F		
**Insecta-Coleoptera**							
Nitidulidae				B			
Curculionidae	F		F				
Dytiscidae							F
Haliplidae							F
Hydrophilidae			F		F	F	F
Elmidae					F		
**Insecta-Hymenoptera**							
Vespidae							F
**Insecta-Hemiptera**							
Gerridae							F
Corixidae	F						F
**Insecta-Dermaptera**							
Forficulidae				S			
**Arthropoda-Arachnida-Araneae**							
Salticidae				F			
**Arthropoda-Arachnida-Acariformes**							
Limnesiidae				F			

### Data analysis

All community data were analyzed and presented using Plymouth Routines in Multivariate Ecological Research package v6 with PERMANOVA+ add-on and STATISTICA version 12. Statistical significance for all analyses was set at *p* < 0.05. To assess whether invertebrate assemblages on submerged and floating mats from the mat survey were similar, a two-dimensional principal coordinates ordination (PCoA) was generated to visualize the data using Bray–Curtis similarity coefficients. Similarity Percentages (SIMPER) were also calculated to determine which taxa contributed most to the Bray–Curtis similarity for each mat type and the dissimilarity between them (Clarke & Gorley, 2006). Permutational multivariate analysis of variance (PERMANOVA) was performed on the community data to determine if invertebrate communities differed between sites and mat types (Anderson, Gorley & Clarke, 2008). Site and mat type, nested within site, were both random factors. Since sampling was opportunistic, i.e., only possible when floating mats occurred and restricted to summer months, time could not be incorporated into the analysis as a factor and thus all samples were pooled through time (note: all sampling occurred during summer months). If a significant result was obtained, pair-wise comparisons were performed. Estimates of components of variance are presented for each variable. All community data were square-root transformed prior to the calculation of Bray–Curtis resemblance matrices, and therefore analyses, to reduce the influence of dominant taxa.

For the Wheatley River natural experiment (summer 2014), invertebrate community data were visualized using PCoA, and the contributions of each species to the Bray–Curtis similarity index were compared using SIMPER. Community data were analyzed using PERMANOVA but with sampling time and mat type as the factors of interest. Initial analysis resulted in a negative estimate for the component of variation and a large *p*-value (*p* = 0.855) for sampling time. As per Underwood (1997) and Anderson, Gorley & Clarke (2008) this term was pooled with the nested term for mat type and sampling time. If main effects were significant, they were again examined using pair-wise comparisons. Comparison was only possible when both mat types were present which occurred in four of the six sampling times. Group average hierarchical CLUSTER analysis coupled with similarity profile routine (SIMPROF) were used to explore how the community responded to the hypoxic event (Clarke & Gorley, 2006).

To assess the impact of hypoxia on invertebrate abundances on submerged and floating mats animals incapable of moving to floating mats of their own volition were eliminated from the community. Thus, the following “mobile” taxa for this analysis included only: amphipods, *Gammarus. mucronatus* Say, 1818, *G. oceanicus* Segerstråle, 1947, *G. lawrencianus* Bousfield, 1956 (Bousfield, 1973), Corophiidae, Ampithoidae (Bousfield, 1973), an isopod: *Jaera* sp. (Pavia, Carr & Aberg, 1999) and larval chironomid flies: Chironomini and Orthocladiinae (Merritt, Cummins & Berg, 2008). To assess the response of mobile taxa to hypoxia, taxa from both years and all sites, were analyzed separately from benthic fauna. Thus, if all mobile animals were on floating mats they would make up 100% of the total population, if mobile animals were equally divided between mat types then they would be 50% of the total population. Specifically, the percentage of mobile taxa on floating mats, relative to the total number of mobile animals from both mat types, was calculated and regressed against the number of hours that were hypoxic (<2 mg/L) over the 48 h preceding sampling. A first order equation was selected to model the relationship between the percentage of mobile invertebrates on floating mats and hypoxia. This function was chosen over other common asymptotic functions as it could incorporate zero hypoxia values without further transformation. As occupancy cannot exceed 100%, the asymptote was forced to 100% as follows:

\begin{eqnarray*}\text{%} \text{invertebrates on floating mats}=\text{%} \text{at zero hypoxia}+(100-\text{%} \text{at zero hypoxia})\quad \times ~ \left( 1-{e}^{(-{k}_{\mathrm{O}}\times \text{Hypoxic hours})} \right) \end{eqnarray*}

where *k*_O_ is a constant describing the rate of migration with hypoxic hours. Non-linear curve fitting was performed using STATISTICA V.12 using the Quasi–Newton estimation method with the standard loss function (Observed-Predicted)^2^.

## Results

### Occupation of submerged and floating mats

**Figure 2:**
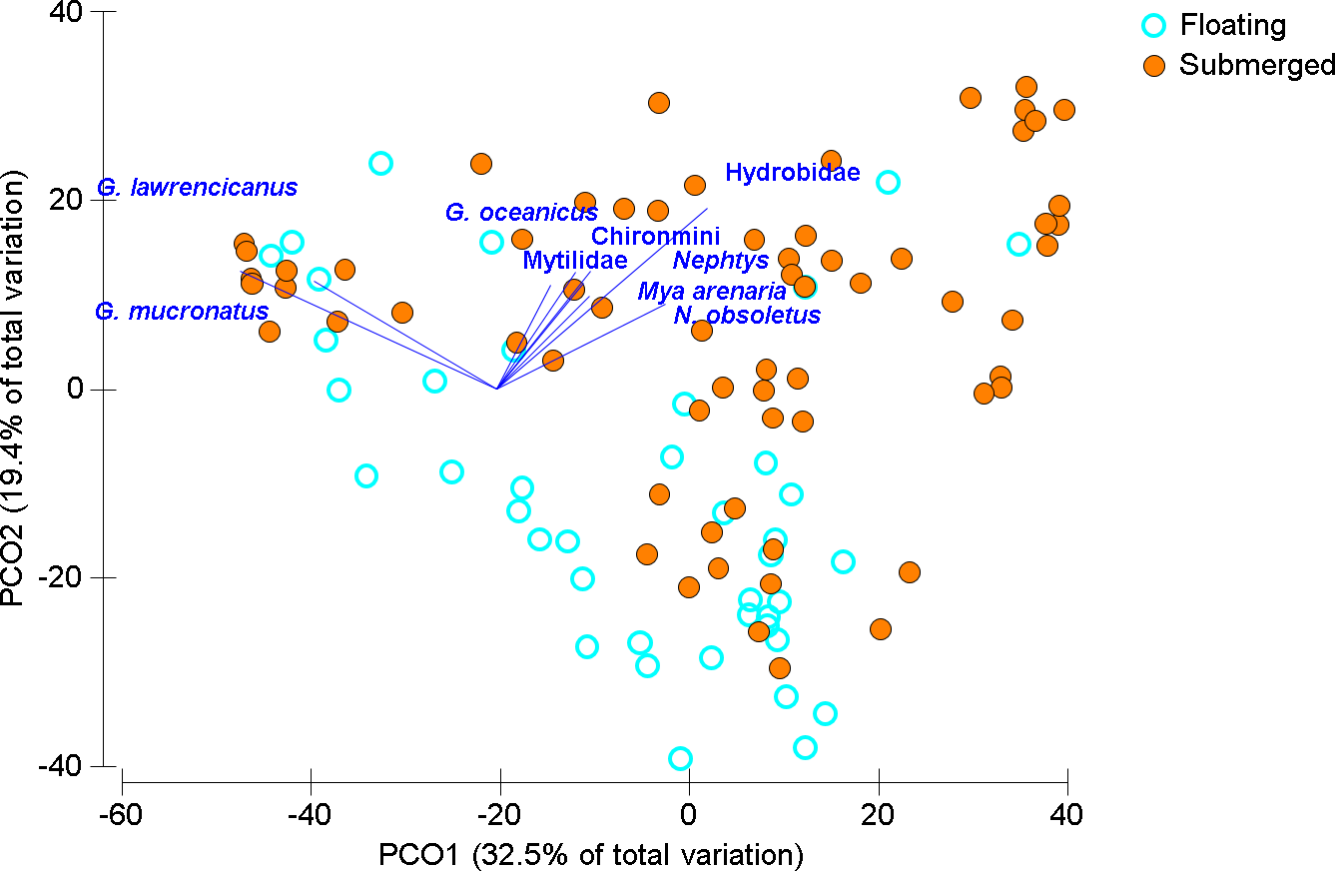
Ordination of invertebrate community data for floating and submerged mats from all sites. Principal Coordinate Ordination for the floating mat survey, seven estuaries discriminated by mat type. Each circle is the Bray-Curtis similarity from a sample, squareroot transformed, sampling times and sites are not distinguished here. Vector length corresponds to the magnitude of the coefficient, which in linear combination with the other variables makes up the axis. The cutoff for this correlation coefficient was set to *r* < 0.35.

Over all sampling times, years, and locations, floating *Ulva* spp. mats were generally present (63% of time/place observations overall). Sites visited at times outside the regular sampling regime indicated that the increased presence of floating mats coincided with hypoxia (summer months), in comparison to spring or fall. Macroinvertebrate communities were statistically distinct between submerged mats (*n* = 65) and floating mats (*n* = 41) though with considerable overlap when visualized using PCoA (Fig. 2 and Table 3). Hydrobid snails and the amphipod *Gammarus mucronatus* were both at highest abundances on submerged mats but also contributed most to the similarity within floating mats, but in reverse order (Table 4). Submerged mats had greater incidence of benthic fauna (e.g., gastropods, bivalves and polychaetes) whereas other taxa such as insects were typically found on floating mats (Tables 2 and 4). Ephydridae larvae, that are typical of salt marshes and feed on decomposing algae (Foote, 1995), were found on the floating mats at all sites except Mill River, which was only sampled once (Tables 1 and 2). Chironomid larvae that are often found in estuaries (Norkko, Bonsdorff & Norkko, 2000) were found on both submerged and floating mats except in Montague where they were only found on floating mats (Table 2). Dissimilarity between mat types was driven by both the higher abundances found on submerged mats and unique species that were only found on floating mats (Tables 2 and 4), although dominant species were generally present in both mat types (Table 4).

**Table 3:** Statistical analyses (PERMANOVAs) for community data. PERMANOVAs were performed on Bray-Curtis distances, abundances were square-root transformed prior to analysis, for invertebrate communities sampled in the (1) Mat Survey and (2) Natural Experiment. For the former, “Site” and “Mat Type (Site)” were random factors. For the Natural Experiment, “Time” and “Mat Type (Time)” were pooled (see ‘Data analysis’).

	PERMANOVA						
	Source	*df*	SS	MS	Pseudo-F	*p*	Estimate of components of variation
^a^Mat Survey (2013)	Site	6	69,836	11,639	2.7734	**0.008**	23.029
	Mat type (site)	7	29,923	4274.7	5.5301	**0.001**	22.089
	Residuals	92	71,116	773			27.803
	Total	105	1.81 e–5				
^b^Natural experiment (2014)	Mat type (time)	9	32,280	3586.7	8.9028	**0.001**	25.234
	Residuals	40	16,115	402.88			20.072
	Total	49	48,395				

**Notes.**

Column headings are standard for an ANOVA design:

*df* degrees of freedom SS Sum of Squares MS Mean Square Pseudo-F is the permutated F-statistic *p* significance level

a No two sites were significantly different, at each site floating ≠ submerged mats.

b At each time floating ≠ submerged mats.

**Table 4:** List of species which contribute most to the Bray-Curtis dissimilarity matrices for the 2013 Mat Survey and the 2014 Natural Experiment. Top ten taxa contributing to the similarity within and dissimilarity between floating and submerged mats (SIMPER analysis) for (1) the Mat Survey and (2) the Natural Experiment. Taxa shown in bold type indicate those whose highest average abundances (individuals/g) occurred in the mat type shown (abundances are back-transformed from the square root data).

	Floating mats			Submerged mats			Contrasting taxa:	
	Avg. similarity within floating mats = 35.98%			Avg. similarity within submerged mats = 36.95%			Avg. dissimilarity between mat types = 67.86%	
	Taxon	Average abundance	Contribution %	Taxon	Average abundance	Contribution %	Taxon	Contribution %
Mat Survey (2013)	*G. mucronatus*	1.21	34.7	**Hydrobiidae**	**2.53**	**36.94**	*G. lawrencianus*	14.56
	Hydrobiidae	1.32	31.7	***G. mucronatus***	**2.07**	**25.51**	Hydrobiidae	13.99
	***G. lawrencianus***	**3.1**	**21.17**	*G. lawrencianus*	**2.16**	**15.1**	*G. mucronatus*	8.12
	Corophiidae	0.19	7.1	**Corophiidae**	0.69	7.4	Corophiidae	6.20
	Mytilidae	0.03	1.4	***M. arenaria***	**0.1**	**2.92**	*Littorina* spp	3.2
	Orthocladiinae	**0.01**	**1.0**	***N. obsoletus***	**0.06**	**2.67**	Mytilidae	2.65
	*Littorina* spp.	0.02	0.57	***Littorina* spp.**	**0.14**	**2.24**	Cerithiidae	2.28
	**Nereidae**	**0.01**	**0.51**	**Cerithiidae**	**0.09**	**1.79**	*M. arenaria*	2.23
	***G. oceanicus***	<**0.001**	**0.21**	**Mytillidae**	**0.14**	**1.26**	*N. obsoletus*	1.98
	**Ephydridae**	<**0.001**	**0.21**	Chironmini	0.09	1.1	***G. oceanicus***	1.91

	Floating mats			Submerged mats			Contrasting taxa:	
	Avg. similarity within floating mats = 58.62%			Avg. similarity within submerged mats = 58.50%			Avg. dissimilarity between mat types = 53.38%	
Natural Experiment (2014)	***G. mucronatus***	**5.24**	**34.95**	***Littorina* spp.**	**10.76**	**39.86**	*Littorina* spp.	20.63
	**Hydrobiidae**	**6.65**	**32.81**	Hydrobiidae	4.93	24.85	*G. mucronatus*	16.51
	*Littorina* spp.	2.86	17.45	***G. lawrencianus***	**2.34**	**12.2**	Hydrobiidae	16.26
	*G. lawrencianus*	0.92	11.05	*G. mucronatus*	0.41	7.1	*G. lawrencianus*	11.62
	Nereidae	0.02	1.08	**Cerithiidae**	**0.74**	**5.57**	Cerithiidae	7.39
	Corophiidae	0.01	0.42	**Pyramidellidae**	**0.19**	**4.13**	Pyramidellidae	4.37
	**Nitidulidae**	**0.01**	**0.17**	***Jaera*** **sp.**	**0.23**	**2.21**	*Jaera* sp.	4.32
	*Jaera* spp.	0.01	0.16	Chironomini	0.03	1.04	Corophiidae	2.50
	*N. obsoletus*	<0.001	0.06	***G. oceanicus***	**0.04**	**0.71**	*G. oceanicus*	2.25
	Pyramidellidae	<0.001	0.02	***N. obsoletus***	**0.01**	**0.67**	Nereidae	1.83

### Community response to anoxia

**Figure 3:**
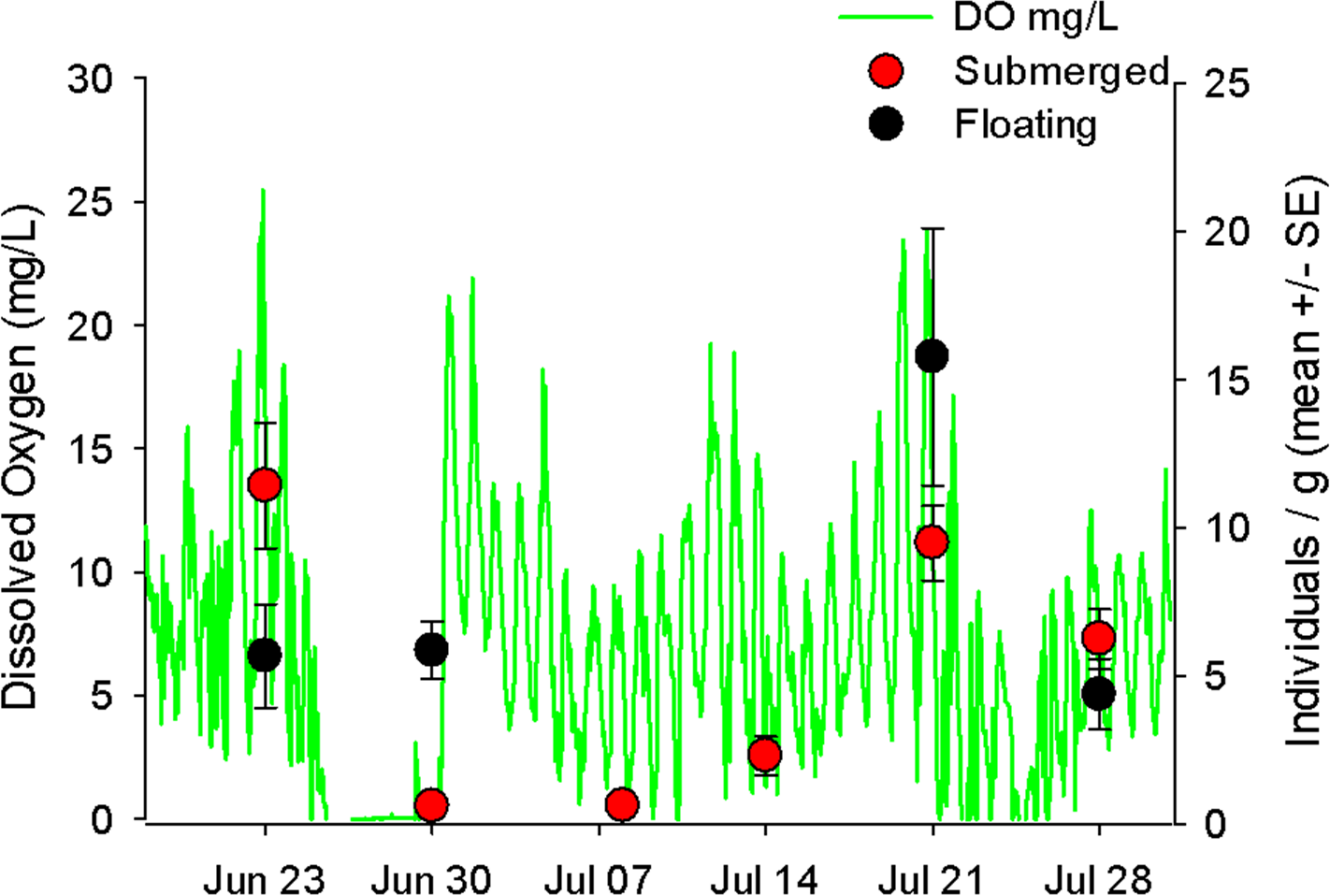
Hourly dissolved oxygen concentration (mg/L) with mobile invertebrates on floating and submerged mats. Hourly dissolved oxygen concentration (mg/L) represented as the green line. On each sampling date are the average number of mobile taxa (±1 S.E.) of five replicates for either submerged (red circles) or floating (black circles) mats.

**Figure 4:**
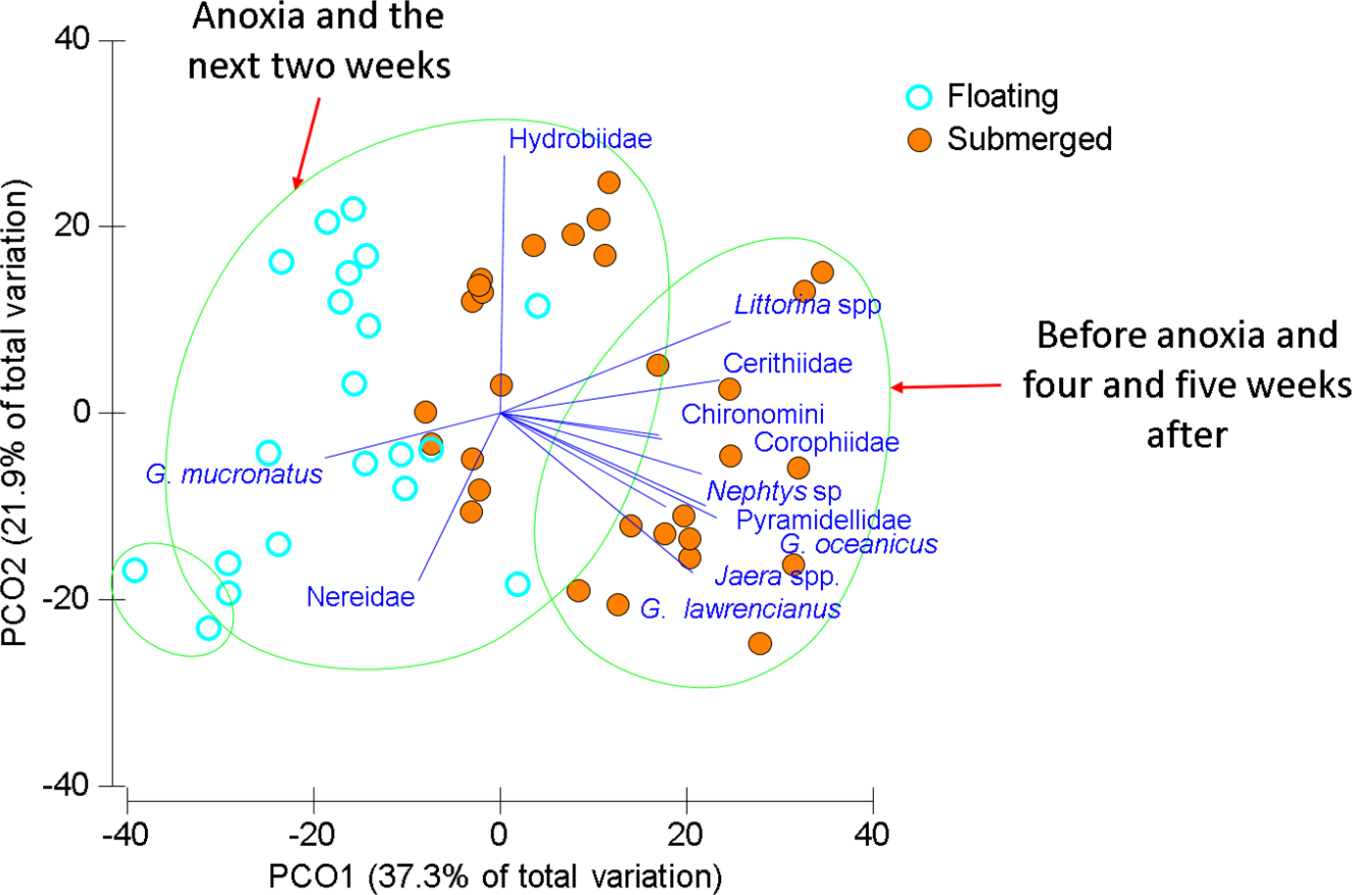
Ordination of invertebrate community data for floating and submerged mats from Wheatley River. Principal Coordinate Ordination for the 2014 Natural Experiment in Wheatley River, discriminated by mat type. Each filled circle is the Bray-Curtis similarity from a sample, squareroot transformed, and statistically significant groups encircled according to a CLUSTER analysis at 60% similarity. Vector length corresponds to the magnitude of the coefficient, which in linear combination with the other variables makes up the axis. The cutoff for this correlation coefficient was set to *r* < 0.5.

In the Wheatley River (2014), dissolved oxygen concentration was high during the first sampling period and then was anoxic for 113 consecutive hours prior to the second sampling period. Qualitative examination of the mobile taxa showed submerged mat abundances started high, decreased during anoxia and over the subsequent two weeks and eventually increased upon return to pre-anoxic levels in the last two weeks (Fig. 3). As with the 2013 mat survey data, the invertebrate communities found on submerged and floating mats in the Wheatley River in 2014 appeared distinct (Fig. 4). Multivariate analysis of the Bray–Curtis resemblance matrix using PERMANOVA again supports the ordination with mat types being significantly different from one another (Table 3). Pair-wise comparisons of the different mat type communities, nested within sampling time (*n* = 4), were significant for all cases (Table 3), i.e., the floating mat community is distinct from the submerged mat community.

Littorinid and hydrobid snails contributed most to the similarity within submerged samples and the amphipod *G. mucronatus* and hydrobid snails contributed most within floating samples (Table 4). Differences between mat types were driven by those same species but also *G. lawrencianus*, Cerithiidae, Pyramidellidae, and *Jaera* sp. (Table 4). As with the mat surveys, various insect species were periodically found on floating mats including some arthropods that are generally associated with terrestrial habitats, including Curculionidae (true weevils or snout beetles) and Salticidae (jumping spiders; Table 2).

Two main groups emerged using CLUSTER analysis on the Bray–Curtis resemblance matrix differentiated by sampling time and mat type (Fig. 4). One large group consisted of most of the floating mats and submerged mats from the two weeks after anoxia, another group was comprised of only floating mats, and a group including submerged mats from the first and last two sampling times. Groups were significantly different from one another beyond 60% similarity using SIMPROF tests (Fig. 4). Generally, submerged samples impacted by anoxia had reduced abundances and diversity whereas floating mats, regardless of dissolved oxygen concentration, had some mobile taxa and fewer benthic invertebrates (Fig. 4 and Table 4).

### Mobile taxa and anoxia

**Figure 5:**
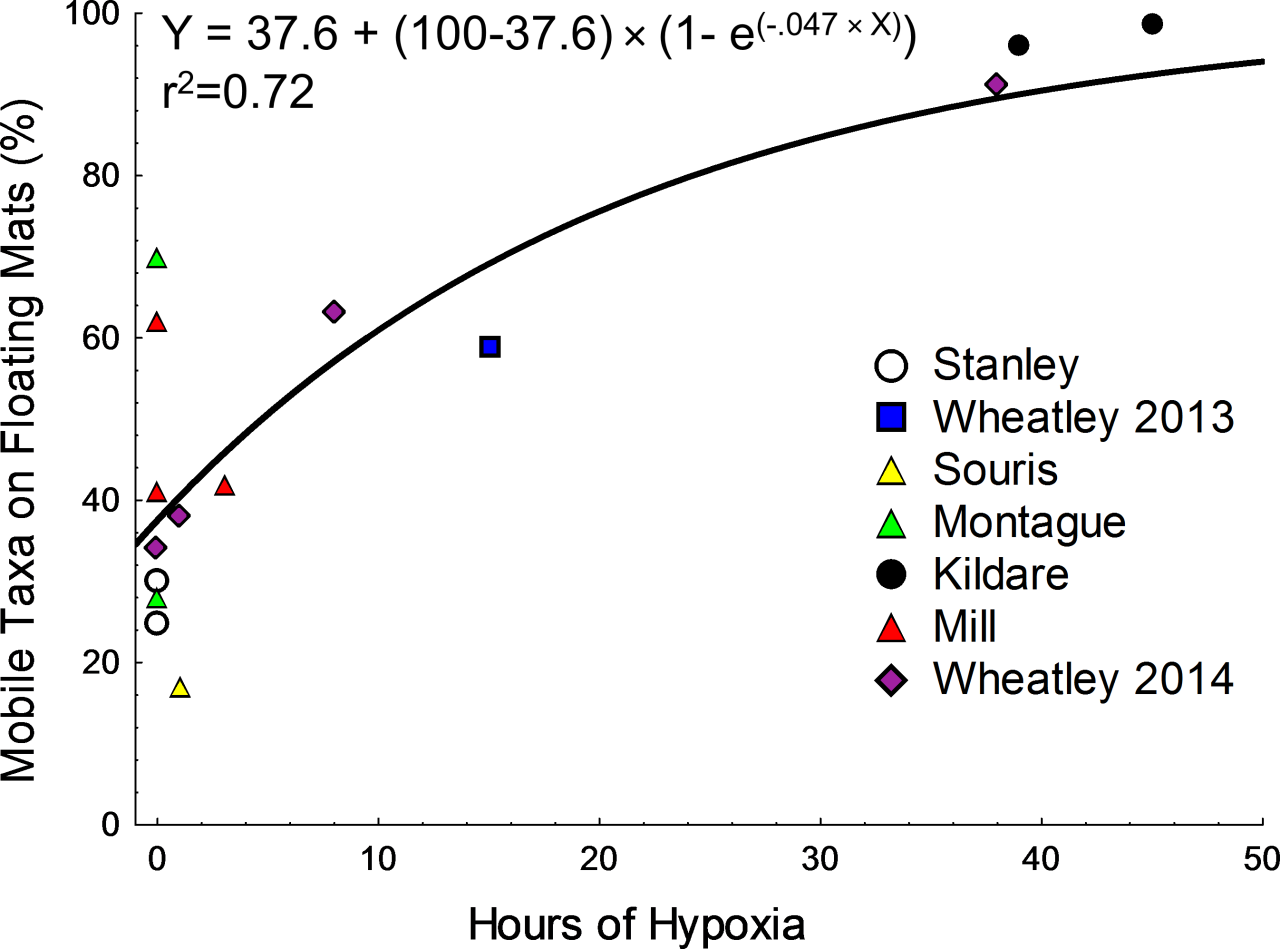
Non-linear regression for the percentage of mobile taxa on floating mats versus the number of hours that were hypoxic in the 48 h preceding sampling. Data (2013 and 2014) were fit using a first order rate equation but forced to an asymptote at 100% (equation and r2 on figure). An error with the dissolved oxygen logger in Murray River forced the exclusion of those data.

The percentage of mobile animals on floating mats, relative to total mobile animal abundances, increased exponentially to an asymptote near 100% with the number of hours that were hypoxic in the 48 h preceding sampling (*r*^2^ = 0.72; Fig. 5). However, this relationship was largely driven by the three sampling events preceded by more than 35 h of hypoxia where the vast majority of individuals were found on floating mats (Fig. 5). Conversely, the percentage of individuals on floating mats during normoxic conditions was closer to those found on submerged mats, with values below 50 meaning that there were a relatively greater number of individuals on submerged mats. These findings support initial observations where amphipods swam more and aggregated on floating mats during periods of hypoxia/anoxia.

In the Wheatley River natural experiment (2014) the abundance of mobile taxa on submerged mats declined during an anoxic event and did not recover until two weeks after (Fig. 3). By contrast, abundance on floating mats showed no such decline immediately after the hypoxic event. Unfortunately, no floating mats occurred for the subsequent two weeks (July 7 and 14) so the persistence of these greater numbers on the floating mats could not be measured.

## Discussion

In eutrophic estuaries, ephemeral algal mats and associated epiphytes provide the primary source of structural habitat and are a significant food source for fauna. The results presented herein suggest that floating vegetative mats may also play a key role in mitigating the impact of sustained anoxia on invertebrate communities in estuaries. The invertebrate community on floating mats was distinct from submerged mats. Mobile taxa were prevalent in both habitats and were used to assess the effect of dissolved oxygen on mat occupancy. Although the percentage of mobile taxa found on floating mats was quite variable under normoxic conditions, there was a positive relationship between the duration of hypoxia and the percentage of mobile taxa on floating mats. More frequent monitoring of the invertebrate community at a single site detected evidence of a population crash; this observation coincided with sustained anoxia, followed by a gradual return to the pre-anoxia community after three weeks.

Free-floating ephemeral algal mats can be formed from many different algal species and have been observed in nutrient-impacted estuaries and coastal systems around the world (Duarte, 1995; Valiela et al., 1997; Raffaelli, Raven & Poole, 1998; Hemminga & Duarte, 2000; Burkholder, Tomasko & Touchette, 2007). While *Ulva* spp. mats are slightly negatively buoyant (Merceron & Morand, 2004), two mechanisms that may bring them to the water’s surface, transitioning from submerged to floating mats, are: (1) turbulence from rising or falling tides which is capable of stirring these floating mats from near the substrate to the surface in shallow systems; and (2) gases, such as hydrogen sulfide, carbon dioxide or methane associated with decomposition (Roden & Tuttle, 1992), and/or oxygen from photosynthesis which propel submerged mats to the surface. When floating, additional tidal forces from the superficial freshwater layer, and/or wind forcing, may lead to greater movement and longer float times (Hammann & Zimmer, 2014).

The relationship between algal mats and macroinvertebrates has been well-studied. In areas lacking vegetation like mud or sand-flats, algal mats are structural habitat, a food source, and cover from predators (Ince et al., 2007; Bishop, Coleman & Kelaher, 2010). However, in all habitats, negative impacts emerge when primary production outstrips herbivory leading to a build-up of decomposing organic matter and hypoxia, ultimately eliminating habitat and decreasing faunal abundance (Berezina, 2008; Arroyo et al., 2012). Like in this study, systems where macroalgae like *Ulva* spp. dominate are characterized by long water residence time and placid waters such as the inner portions of microtidal estuaries, coastal lagoons and embayments (Orth et al., 2006; Short et al., 2006; Burkholder, Tomasko & Touchette, 2007). Consequently, submerged *Ulva* spp. mats are not generally considered as a dispersal method for invertebrate fauna. Vertical distribution of macroinvertebrates on submerged mats in shallow estuaries has been studied however (Salovius & Bonsdorff, 2004; Salovius, Nyqvist & Bonsdorff, 2005), although not frequently. Salovius, Nyqvist & Bonsdorff (2005) found a greater incidence of fauna more associated with sediment on algal mats closer to the substrate than higher in the water column, but they did not study mats on the water’s surface explicitly.

For fauna occupying a floating mat, algal or otherwise, there are some inherent risks. In summer, water temperature increases with height in the water column, salinity is typically lower than near bottom, and other physicochemical parameters are more variable (Valiela, 1995). Predation risk is known to decrease with increasing habitat complexity (Russo, 1987; Gehrels et al., 2016) and may also decrease in hypoxic habitats that predators cannot tolerate (Nestlerode & Diaz, 1998). Floating mats are generally smaller and less dense than submerged mats but it is unknown if/how this affects predation risk (Marklund, Blindow & Hargeby, 2001). Furthermore, the fish that might prey upon the macroinvertebrates inhabiting floating mats may not risk feeding higher in the water column to avoid attack by aquatic and avian predators.

The lack of information on floating *Ulva* spp. mats is not surprising as they are not common in all systems and, to our knowledge, not considered rafts. Reviews of taxa that raft in marine systems by Thiel and co-authors (Thiel & Gutow, 2005; Thiel & Haye, 2006) provide excellent examples of the prevalence and the evolutionary and dispersal implications of rafting in general. It has been demonstrated that species, such as amphipods, frequently found on eelgrass wrack are also found on floating *Ulva* spp. mats (Thiel & Gutow, 2005; Thiel & Haye, 2006; Vandendriessche, Vincx & Degraer, 2006). Thus, as seagrass is displaced by macroalgae floating seagrass wrack may also be functionally replaced by ephemeral algal mats at the water’s surface. Whether seagrass or algae, wrack is a critical component of dune and beach habitat for fauna (Ince et al., 2007; Olabarria, Lastra & Garrido, 2007; MacMillan & Quijón, 2012). Given global declines in seagrass coverage, it is plausible that macroalgae mats could help mitigate the effects of reduced wrack in those habitats as well.

The mechanism through which mobile macro-invertebrates get to floating mats remains unclear but two possibilities are likely: (1) hypoxic or anoxic conditions elicit a swimming response to acquire oxygen near the surface (Haselmair et al., 2010); and/or (2) taxa on submerged mats that become floating mats stay there during hypoxia. In either case it appears that mobile taxa, at least, benefit from the occurrence of floating mats during hypoxia.

There are many examples of refugia in both terrestrial and aquatic ecosystems (Keppel et al., 2012), but they are typically temporally and spatially stable and encompass larger scale areas. In the Berg River Estuary, a hydrogen sulfide “black tide” from the decomposition of algae, created anoxic conditions throughout the majority of the estuary leading to massive fish mortality (Lamberth, Branch & Clark, 2010). However, the authors noted that some fish capable of tolerating more variable physicochemical conditions were able to avoid the “black tide” by entering the refugium of the upper estuary (Lamberth, Branch & Clark, 2010). The upper portions of nutrient-impacted PEI estuaries are actually the reverse, with more frequent and sustained hypoxia than exists in either the outer estuary or upstream freshwater areas (Bugden et al., 2014). In these areas, complete mortality of infauna from anoxia, and presumably hydrogen sulfide, has been observed at some of our study sites but impacts did not extend beyond the upper estuary (M Coffin, pers. obs., 2013). The present study indicates that hypoxia must be sustained for at least one day before significant impacts are observed, but it is unclear where the thresholds for duration, severity and the spatial extent of hypoxia lie (see Haselmair et al., 2010). Perhaps due to the intensity and frequency of anoxic events at most of our study sites, there were fewer hypoxia sensitive species excepting the mobile taxa which are all crustaceans. Thus, only relatively mobile or hypoxia tolerant animals were ever sampled in this study. This differs from the results of Tweedley et al. (2016) that showed a shift in the community from hypoxia sensitive to hypoxia tolerant species, i.e., a loss of crustaceans and relative increase of annelids, after storm-induced hypoxia.

Typically dissolved oxygen is lowest near the substrate (Valiela, 1995), where the majority of animals live, so occupying rising or floating mats will result in exposure to higher dissolved oxygen concentration. Being higher in the water column is not the only advantage, however, as these mats move either upstream or downstream depending on the prevailing winds and currents. Thus, there are two clear advantages of occupying floating mats: higher dissolved oxygen concentration at the water’s surface, and the potential to be relocated to a less affected area. Therefore, any opportunity for fauna to escape the affected area would be expected to have positive implications for recovery.

Interestingly, the Wheatley River invertebrate community appeared to recover from the 2014 hypoxia/anoxia event after five weeks. The abundance of fauna on floating mats during this event was much less variable than were numbers on submerged mats. These data suggested that, although variable, some members of the invertebrate community can persist on floating mats despite water-column hypoxia. Furthermore, the implication is that mobile taxa may not exhibit a preference for floating mats during hypoxia, but those already on mats are still able to escape by chance.

It is unclear what happens to the submerged mat community during sustained hypoxia. Certain benthic taxa such as gastropods, bivalves and polychaetes are relatively resilient to low oxygen (Miller, Poucher & Coiro, 2002; Riedel et al., 2014), and their densities were found to be similar across sampling times and hypoxic conditions. The presence of mobile taxa on floating mats and not on submerged mats during hypoxia might be explained by mortality, emigration or some combination of both. However, we have no evidence for the first possibility, as dead invertebrates were never found in the submerged mats during sampling. Observations in the field did suggest that amphipods swam more during periods of oxygen stress but overall animal abundances did not increase as expected on the floating mats. Regardless, the community seemed to recover after a relatively short period of time. For gammarid amphipods, sampling frequency was shorter than the duration of embryonic development in the brood pouch had there been reproduction (Steele & Steele, 1974) and no significant growth (Vassallo & Steele, 1980) was observed. Although not measured explicitly, gammarids appeared to be from the same cohort, indicating that the affected area was repopulated via immigration and not internal dynamics. It is likely that the majority of animals that re-colonize the unoccupied/impacted area do so through typical dispersal means, but they may be supplemented by invertebrates occupying floating algal mats (Highsmith, 1985; Arroyo, Aarnio & Bonsdorff, 2006; Lamberth, Branch & Clark, 2010). There is limited evidence that some of the floating mats sampled were from outer or upper estuary locations relative to the sampling area. A few species of small snails, *Bittium* sp. and *Astyris lunata* common in seagrass that only occurs downstream in these estuaries, and hydrophilidae beetles, that are more typical of fresh or brackish water, were found in our collections from floating mats (Barnes, 1994). Additionally, terrestrial taxa may be found on originally submerged mats at low tide (if the tide is low enough to expose the submerged mat to the surface), as indicated by terrestrial sap beetles (Nitidulidae) found in submerged mat collections.

Eutrophication negatively affects estuarine habitat, altering its structure and overall ecosystem quality (Duarte, 1995; Valiela et al., 1997). However, this study indicates that the impact of the most severe symptoms of eutrophication, the loss of seagrass habitat and hypoxia, may be somewhat mitigated by the algae that cause them. Algae provide some of the ecosystem services provided by seagrasses and seagrass wrack. Most significantly, some animals can escape hypoxia/anoxia and, possibly, return via floating mats. Overall, any acceleration of community recovery is important for mitigating the otherwise negative effects of hypoxia. Further research is needed to elucidate the degree to which floating mats act as refugia and if they are a significant mechanism for community recovery after sustained hypoxia.

##  Supplemental Information

10.7717/peerj.3080/supp-1 Data S1 Data used for figure generation and analyses S1 - Raw data for taxa sampled, along with sampling month and estuary for the 2013 mat survey (Table 1) and the 2014 natural experiment.

## Additional Information and Declarations

### Data Availability

The following information was supplied regarding data availability: The data contained within this manuscript is included within the tables and figures, and has been supplied as a  Supplementary File.
